# Health‐related quality of life in patients with esophageal cancer or precancerous lesions assessed by EQ‐5D: A multicenter cross‐sectional study

**DOI:** 10.1111/1759-7714.13368

**Published:** 2020-03-04

**Authors:** Youqing Wang, Jufang Shi, Lingbin Du, Huiyao Huang, Le Wang, Juan Zhu, Huizhang Li, Yana Bai, Xianzhen Liao, Ayan Mao, Guoxiang Liu, Jiansong Ren, Xiaojie Sun, Jiyong Gong, Qi Zhou, Ling Mai, Lin Zhu, Xiaojing Xing, Yuqin Liu, Ying Ren, Bingbing Song, Li Lan, Jinyi Zhou, Peian Lou, Xiaohua Sun, Xiao Qi, Shouling Wu, Wenqiang Wei, Kai Zhang, Min Dai, Wanqing Chen, Jie He

**Affiliations:** ^1^ Institute of Cancer and Basic Medicine (ICBM), Chinese Academy of Sciences/Department of Cancer Prevention, Cancer Hospital of the University of Chinese Academy of Sciences/Department of Cancer Prevention Zhejiang Cancer Hospital Hangzhou China; ^2^ Office of Cancer Screening, National Cancer Center/National Clinical Research Center for Cancer/Cancer Hospital Chinese Academy of Medical Sciences and Peking Union Medical College Beijing China; ^3^ Institute of Epidemiology and Health Statistics Lanzhou University Lanzhou China; ^4^ Hunan Office for Cancer Control and Research Hunan Provincial Cancer Hospital Changsha China; ^5^ Public Health Information Research Office, Institute of Medical Information Chinese Academy of Medical Sciences Beijing China; ^6^ Department of Health Economics, School of Health Management Harbin Medical University Harbin China; ^7^ Center for Health Management and Policy, Key Lab of Health Economics and Policy Shandong University Jinan China; ^8^ Science and Education Department of Public Health Division Shandong Tumor Hospital Jinan China; ^9^ Chongqing Office for Cancer Control and Research Chongqing Cancer Hospital Chongqing China; ^10^ Department of Institute of Tumor Research Affiliated Cancer Hospital of Zhengzhou University, Henan Cancer Hospital Zhengzhou China; ^11^ Teaching and Research Department Affiliated Cancer Hospital of Xinjiang Medical University Urumqi China; ^12^ Liaoning Office for Cancer Control and Research Liaoning Cancer Hospital & Institute Shenyang China; ^13^ Cancer Epidemiology Research Center Gansu Provincial Cancer Hospital Lanzhou China; ^14^ Urban Office of Cancer Early Detection and Treatment Tieling Central Hospital Tieling China; ^15^ Heilongjiang Office for Cancer Control and Research Affiliated Cancer Hospital of Harbin Medical University Harbin China; ^16^ Institute of Chronic Disease Prevention and Control Harbin Center for Disease Control and Prevention Harbin China; ^17^ Institute of Chronic Non‐communicable Diseases Prevention and Control Jiangsu Provincial Center for Disease Control and Prevention Nanjing China; ^18^ Department of Control and Prevention of Chronic Non‐Communicable Diseases Xuzhou Center for Disease Control and Prevention Xuzhou China; ^19^ Ningbo Clinical Cancer Prevention Guidance Center Ningbo No.2 Hospital Ningbo China; ^20^ Department of Occupational Medicine Tangshan People's Hospital Tangshan China; ^21^ Health Department of Kailuan Group Kailuan General Hospital Tangshan China

**Keywords:** EQ‐5D‐3L, esophageal cancer, health state utility, health‐related quality of life, multivariable linear regression

## Abstract

**Background:**

We aimed to obtain a set of health state utility scores of patients with esophageal cancer (EC) and precancerous lesions in China, and to explore the influencing factors of health‐related quality of life (HRQoL).

**Methods:**

A hospital‐based multicenter cross‐sectional study was conducted. From 2013 to 2014, patients with EC or precancerous lesions were enrolled. HRQoL was assessed using a European quality of life‐5 dimension (EQ‐5D‐3L) instrument. Multivariable linear regression analysis was performed to explore the influencing factors of the EQ‐5D utility scores.

**Results:**

A total of 2090 EC patients and 156 precancer patients were included in the study. The dimension of pain/discomfort had the highest rate of self‐reported problems, 60.5% in EC and 51.3% in precancer patients. The mean visual analog scale (VAS) score for EC and precancer patients were 68.4 ± 0.7 and 64.5 ± 3.1, respectively. The EQ‐5D utility scores for EC and precancer patients were estimated as 0.748 ± 0.009 and 0.852 ± 0.022, and the scores of EC at stage I, stage II, stage III, and stage IV were 0.693 ± 0.031, 0.747 ± 0.014, 0.762 ± 0.015, and 0.750 ± 0.023, respectively. According to the multivariable analyses, the factors of region, occupation, household income in 2012, health care insurance type, pathological type, type of therapy, and time points of the survey were statistically associated with the EQ‐5D utility scores of EC patients.

**Conclusions:**

There were remarkable decrements of utility scores among esophageal cancer patients, compared with precancer patients. The specific utility scores of EC would support further cost‐utility analysis in populations in China.

## Introduction

Esophageal cancer (EC) is one of the most prevalent malignant tumors in the upper digestive system. According to GLOBOCAN 2018, it is the seventh most common cancer and the sixth most common cause of death from cancer worldwide, and the estimated numbers of EC new cases and deaths in 2018 were 572 034 and 508 585, respectively.^1^ In China, EC is the sixth most prevalent cancer type, affecting approximately 17.87 per 100 000 individuals. With a mortality rate of 13.68 per 100 000 people, it has been ranked fourth as a leading cause of cancer death in 2015.^2^ The prognosis of EC is poor, with a five‐year relative survival of 20.9% in China.^3^ The postoperative prognosis of patients with early EC is much better, with a five‐year survival rate of ≥90% as reported in the study by Sadiq and Mansour.^4^ However, most early EC or precancerous lesions which show no typical clinical symptoms cannot be easily detected.^5^ As the risk of EC development can be reduced after the removal of precancerous lesions and the prognosis improves,^6^ early detection and treatment is particularly important. Although regular follow‐up has been recommended for most patients with early EC and precancerous lesions, the compliance of patients undergoing long‐term follow‐up visits is relatively poor, which has led to a considerable psychological burden for these patients.^5^ Furthermore, previous studies have indicated that the incidence rates of EC have decreased in China over the last 20 years, but EC has still been a significant sociopsychological and economic burden for patients.^7^ It is of great significance to understand EC or precancer patients' health‐related quality of life (HRQoL), which will also benefit healthcare services.

Quality of life (QoL) has been one of the critical indicators in cost‐utility analysis in health economic evaluation.^8^ QoL reflects one's subjective perceptions, goals, expectations and concerns in relation to his or her living environment,^9^ meanwhile HRQoL is a subjective assessment of health status.^10^ It is common to include HRQoL measures in oncology.^11^ HRQoL has been recognized as an important measure of assessing the outcome of diagnosis and the impact of cancer treatments on patients.^12^, ^13^ Health state utility (HSU) is a measure of preference‐based HRQoL often used by health economists, and it is unique because it represents an individual's valuation or preference for being in a particular health state.^8^ The European quality of life‐5 dimension (EQ‐5D) is an indirect measure of utility for health that generates an index‐based summary score based upon societal preference weights,^14^ which has been used to assess therapeutic benefits^15^ and in healthcare surveys across diverse general populations.^16^, ^17^ The EQ‐5D‐3L, which is the original version of the EQ‐5D with five dimensions and three levels on each dimension, has been widely applied to measuring general health conditions.^18^, ^19^, ^20^, ^21^, ^22^, ^23^, ^24^, ^25^ However, in previous EC studies, due to the absence of an EQ‐5D preference weight set in the Chinese population, Chinese researchers usually tended to choose the English preference weight set to estimate the EQ‐5D utility scores.^26^, ^27^ In 2014, Liu *et al*. successfully developed Chinese utility values for EQ‐5D‐3L health states using the time trade‐off method.^28^ Therefore, we adopted the Chinese general population‐based algorithm in the current study.

In recent years, HRQoL has become a major measurement of clinical research. Several studies have measured the HRQoL directly in EC patients.^29^, ^30^, ^31^ Liu *et al*. reported EC significantly impaired Chinese patients' HRQoL in daily life after treatment^32^; Lin *et al*. reported that personal characteristics were associated with patients' HRQoL in China.^33^ More studies from abroad focused on the impact of surgery or other treatments on the HRQoL of EC patients.^34^, ^35^, ^36^ However, as far as we know, published empirical studies on the health condition effects and accurate data on the HRQoL utility values of EC and precancer in China remain scarce. In past decades, early detection and treatment of EC provided the best opportunity for cure.^37^ To justify the cost of cancer screening techniques or therapeutic methods, policy‐makers need to determine if there is statistical significance of cancer screening methods for cancer survival or the HRQoL of patients.

The objectives of our study were as follows: (i) To obtain a set of health state utility scores of patients with EC and precancerous lesions in China using the EQ‐5D instrument, and (ii) to evaluate the determinants of HRQoL and the relationship between these influencing factors and EQ‐5D utility.

## Methods

### Study design

The National Cancer Center of China conducted a hospital‐based multicenter cross‐sectional study, namely the Cancer Screening Program in Urban China (CanSPUC), which provides free screening services for Chinese urban residents aged from 40–69 years. The target cancers in this screening program were lung, breast, colorectal, liver, stomach and esophageal cancers. It was a major public health service project supported by the central government of China. This project was initiated in August 2012, and has now covered 29 provinces nationwide. The screening process consisted of three steps: the initial assessment of a high‐risk population, further screening for cancer, and health economic evaluation.

This current EQ‐5D study was one part of the overall project, CanSPUC. We aimed to use the utility instrument EQ‐5D‐3L to systematically evaluate the HRQoL in patients with EC or precancerous lesions in China. From 2013 to 2014, a total of 12 provinces in China were enrolled in the CanSPUC, which were distributed in four geographic regions (East, Central, West, and Northeast).^38^

### Patient selection

We identified cases of esophageal precancerous lesions as low grade intraepithelial lesions (LSIL) and high grade intraepithelial lesions (HSIL); LSIL included mild dysplasia and moderate dysplasia, and HSIL referred to severe dysplasia and carcinoma, in situ.^39^

In the present study, patients were selected using the method of convenience sampling in each involved medical center. The eligibility criteria for participants were as follows: (i) Patients were 40–69 years of age at the initial diagnosis with EC or precancerous lesions by screening, from September 2013 to December 2014; (ii) residents of the 12 selected provinces; and (iii) able to understand the survey procedure and complete the survey questionnaire. The survey was approved by the Institutional Review Board of the Cancer Hospital of Chinese Academy of Medical Sciences (Approval No. 15‐071/998). Written informed consent was signed before each participant was enrolled into the study.

### Data collection

A total of 2090 patients with EC and 156 patients with precancerous lesions were included in the present study. Each patient was interviewed with a structured questionnaire for sociodemographic, clinical, and HRQoL information. The HRQoL was assessed using the EQ‐5D‐3L questionnaire. In the EQ‐5D questionnaire, respondents were at particular levels in particular dimensions, and the three levels of each dimension were categorized into “no problems” (level 1) and “any problem” (levels 2 and 3).^40^ Utility values were defined on a scale from 0–1, with 0 representing death and 1 representing perfect health. We applied a preference weight set for the Chinese population to estimate the mean EQ‐5D utility score.^28^ In addition, further patient information including sociodemographics and clinical characteristics, such as age, sex, region of residence, education, occupation, marital status, income, health care insurance type, age at diagnosis, pathological type, type of therapy, time points of the survey, and clinical stage for EC according to the seventh edition of the American Joint Committee on Cancer/International Union Against Cancer staging system,^41^ were also collected.

### Quality control

First, study protocol and data collection training were provided by the National Cancer Center of China for all the principle staff from the abovementioned 12 sites. Second, all the participating physicians in local centers/hospitals were trained for in‐person interviewing using a structured questionnaire with an EQ‐5D instrument (Chinese version). A face‐to‐face interview was either administered by a trained interviewer, or alternatively, self‐administered by capable patients in the presence of the project staff member who could answer any doubts the patients had about the interview. Furthermore, data input and basic data checks were also performed in the 15 collaborative sites. The National Cancer Center of China conducted multiple rounds of data logistical checks by interacting with local staff, and was responsible for the database building, data cleaning, and data analyses.

### Statistical analysis

Frequencies and percentages were used for categorical variables, and the mean ± standard deviation (SD) was used for continuous variables. Means of EQ VAS scores and EQ‐5D utility scores were analyzed (overall, by sex and by age), using the Kruskal‐Wallis test, which is appropriate for non‐normal data. The Kruskal‐Wallis test was also used to compare the means of EQ‐5D utility scores across different categories of sociodemographics (region, education, occupation, marital status, household income, health care insurance type, and age at diagnosis) and clinical characteristics (pathological type, type of therapy, and time points of the survey). Finally, we conducted a multivariate linear ordinary least square (OLS) regression analysis to explore the impact of important variables, by inputting variables with statistical significance confirmed in the univariate analysis, and using a stepwise approach. OLS regression analysis is known to be the most commonly used and optimal method for HSU multivariate analyses so far,^42^, ^43^, ^44^, ^45^ and it is sensitive to non‐normal data.^46^ *P*‐values <0.05 were considered statistically significant. All hypothetical tests were two‐sided.

Data were entered into the EpiData software (version 3.1, EpiData Association, Odense, Denmark), using a double entry method. Logical check and statistical analyses were performed using SAS statistical software for Windows, version 9.3 (SAS Institute, Cary, NC, USA).

## Results

### Demographics of the participants

The sociodemographic and clinical characteristics of the patients are summarized in Table 1. The participant rate of EC patients was 87.1% and that of precancer was 90.2%.

**Table 1:** Sociodemographic and clinical characteristics of patients

Variables	Patients with esophageal precancerous lesions (*N* = 156)	Patients with esophageal cancer (*N* = 2090)
Age, mean ± SD	57.29 ± 12.86	62.64 ± 9.06
Age, years, n (%)		
40–44	26 (16.67)	43 (2.06)
45–49	8 (5.13)	118 (5.65)
50–54	23 (14.74)	227 (10.86)
55–59	28 (17.95)	355 (16.99)
60–64	29 (18.59)	490 (23.44)
65–69	42 (26.92)	857 (41.00)
Sex, n (%)		
Male	105 (67.31)	1675 (80.14)
Female	51 (32.69)	415 (19.86)
Region, n (%)		
East	79 (50.64)	823 (39.38)
Central	2 (1.28)	465 (22.25)
West	38 (24.36)	559 (26.75)
Northeast	37 (23.72)	243 (11.63)
Education, n (%)		
Primary school or below	49 (31.41)	1004 (48.04)
Junior high school	58 (37.18)	631 (30.19)
Senior high school	30 (19.23)	333 (15.93)
Undergraduate or over	19 (12.18)	120 (5.74)
Occupation, n (%)^a^		
Farmer	56 (35.90)	1103 (52.78)
Enterprise or company employee/worker	33 (21.15)	290 (13.88)
Self‐employed or unemployed	18 (11.54)	224 (10.72)
Retiree	14 (8.97)	127 (6.08)
Public sector employee	33 (21.15)	341 (16.32)
Other	2 (1.28)	5 (0.24)
Marital status, n (%)		
Married	147 (94.23)	1979 (94.69)
Other	9 (5.77)	111 (5.31)
Household income in 2012, CNY, n (%)^a^		
<20 000	16 (10.26)	469 (22.44)
20 000–	23 (14.74)	563 (26.94)
40 000–	31 (19.87)	513 (24.55)
60 000–	33 (21.15)	271 (12.97)
≥80 000	53 (33.97)	271 (12.97)
Health care insurance type, n (%)		
Urban employee basic medical insurance	61 (39.10)	543 (25.98)
Urban residents basic medical insurance	24 (15.38)	261 (12.49)
New rural cooperative medical scheme	66 (42.31)	1201 (57.46)
Self‐pay	2 (1.28)	19 (0.91)
Other	3 (1.92)	66 (3.16)
Age at diagnosis, years^a^		
<45	26 (16.67)	54 (2.58)
45–54	35 (22.44)	351 (16.79)
55–64	49 (31.41)	794 (37.99)
≥65	38 (24.36)	831 (39.76)
Pathological type^a^		
Low grade squamous intraepithelial tumors	34 (21.79)	—
High grade squamous intraepithelial neoplasms	36 (23.08)	—
Adenoma	—	142 (6.79)
Squamous cell carcinoma	—	1417 (67.80)
Other types of cancer	—	361 (17.27)
Stage^a^		
Stage I	—	194 (9.28)
Stage II	—	761 (36.41)
Stage III	—	679 (32.49)
Stage IV	—	302 (14.45)
Type of therapy^a^		
Surgery	63 (40.38)	611 (29.23)
Symptomatic treatment	62 (39.74)	264 (12.63)
Radiotherapy	—	303 (14.50)
Chemotherapy	—	385 (18.42)
Surgery & postoperative chemotherapy	—	201 (9.62)
Neoadjuvant chemotherapy & surgery	—	42 (2.01)
Concurrent chemoradiotherapy	—	205 (9.81)
Other	—	19 (0.91)
Time points of the survey^a^		
Pretreatment	38 (24.36)	263 (12.58)
In treatment	31 (19.87)	1284 (61.44)
Post‐treatment	51 (32.69)	365 (17.46)
Follow‐up	23 (14.74)	114 (5.45)

^a^The sum of the numbers for some characteristic variables is less than the total due to missing values.

A total of 2090 patients with EC were included in the analysis (1675 males and 415 females), with a mean age of 62.64 ± 9.06 years. Most of the patients were 65–69 years of age and of all the cancer patients in the analysis, 39.38% were from the eastern region. A majority of the EC patients were married (94.69%). Among 1920 patients with available pathological information, 1417 patients were diagnosed with squamous cell carcinoma and 142 patients were diagnosed with adenoma. Among 1936 patients with available stage information, 761 patients were diagnosed with stage II cancer, whereas only 194 had stage I cancer. Of all the EC patients, 611 (29.23%) patients underwent surgery, 385 (18.42%) patients received chemotherapy and 303 (14.50%) patients received radiotherapy.

There was a total of 156 patients with esophageal precancerous lesions (105 males and 51 females), with a mean age of 57.29 ± 12.86 years. The sociodemographic characteristics were similar to those of the EC patients. Among 70 patients with available pathological information, 36 patients were diagnosed with high grade squamous intraepithelial neoplasms and 34 patients with low grade squamous intraepithelial tumors. A total of 63 patients underwent surgery and 62 patients received symptomatic treatment.

### Health status of the participants

**Figure 1:**
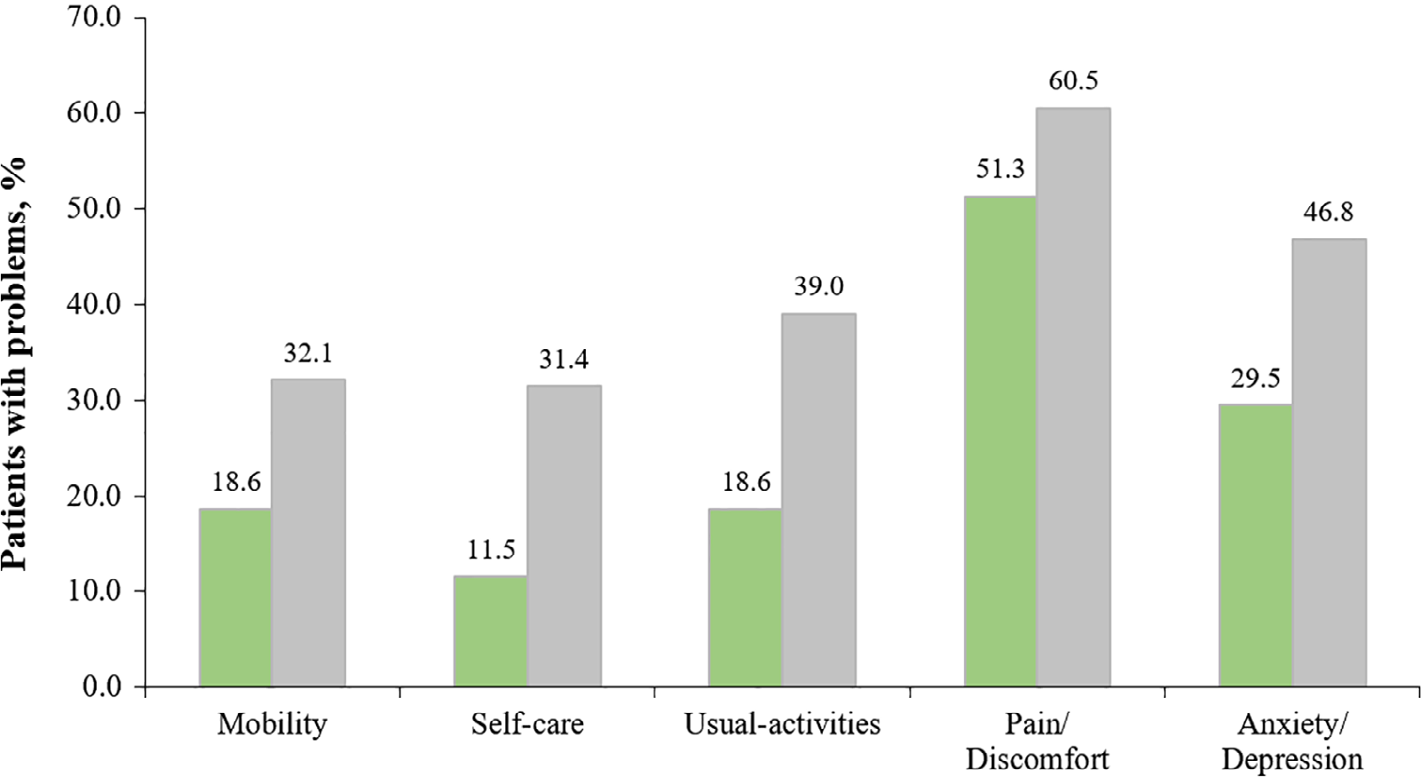
Distribution of self‐reported EQ‐5D problems. (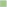) Patients with esophageal precancerous lesions and (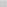) patients with esophageal cancer.

**Figure 2:**
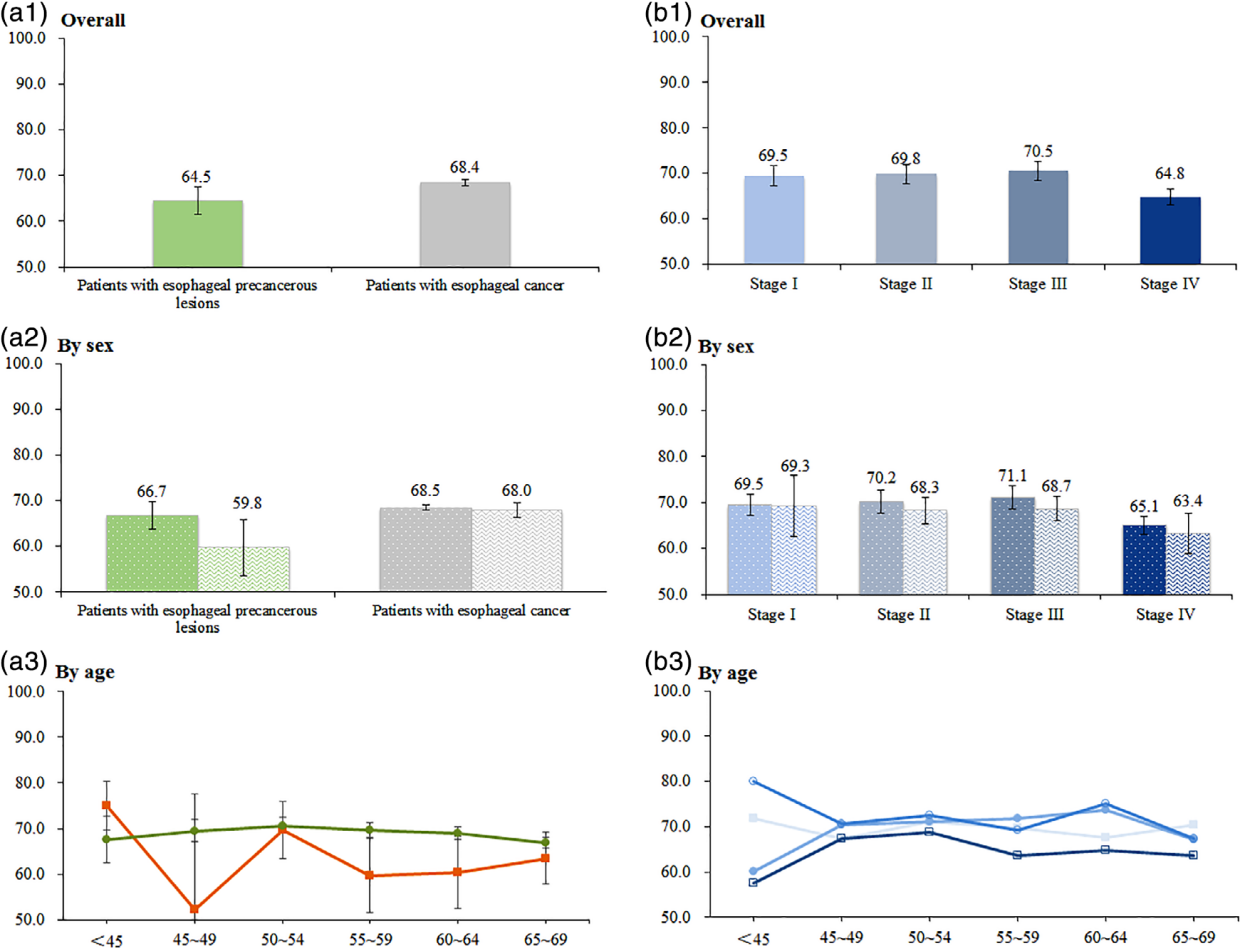
Mean of VAS scores, overall and by sex and age. (**a2**) (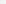) Male and (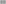) female. (**b2**) (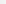) Male and (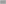) female. (**a3**) (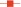) Patients with esophageal precancerous lesions and (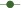) patients with esophageal cancer. (**b3**) (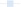) Stage I, (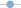) Stage II, (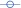) Stage III and (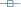) Stage IV.

**Figure 3:**
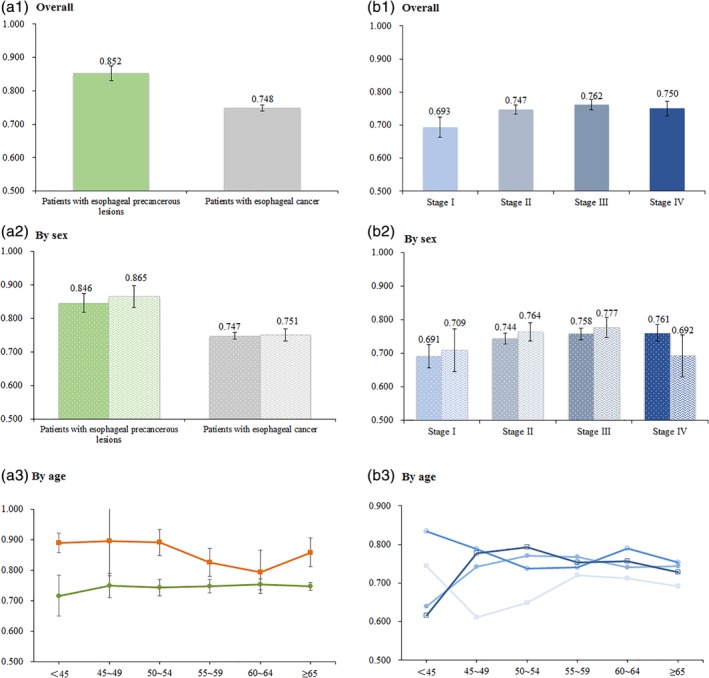
Mean of EQ‐5D index scores, overall and by sex and age. (**a2**) (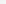) Male and (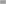) female. (**b2**) (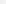) Male and (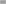) female. (**a3**) (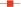) Patients with esophageal precancerous lesions and (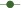) patients with esophageal cancer. (**b3**) (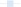) Stage I, (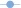) Stage II, (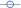) Stage III and (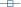) Stage IV.

Figure 1 shows the results of the five dimensions. Compared to the patients with precancerous lesions, cancer patients tended to report more problems in all dimensions, with all *P*‐values <0.05 (Kruskal‐Wallis test results). Figures 2 and 3 present further detailed mean VAS and EQ‐5D utility scores for different subgroups. The overall VAS means among patients with EC and precancerous lesions were 68.4 ± 0.7 and 64.5 ± 3.1 (Fig 2a1), respectively, but there was no significant difference between the two groups. For cancer patients, significant differences of VAS were detected among subgroups by age (Fig 2a3) and by cancer stages (Fig 2b1) (both *P*‐values <0.05). The mean EQ‐VAS scores were 69.5 ± 2.2 at stage I, 69.8 ± 2.1 at stage II, 70.5 ± 2.1 at stage III, and 64.8 ± 1.8 at stage IV. After applying the preference weights of the Chinese population, the overall EQ‐5D utility scores of cancer patients and patients with precancerous lesions were 0.748 ± 0.009 and 0.852 ± 0.022, respectively. Compared with EC patients, patients with precancerous lesions were statistically associated with higher EQ‐5D utility scores (Fig 3a1) (*P* < 0.0001). A significant difference of EQ‐5D utility scores was detected among four cancer stages (Fig 3b1) (*P* = 0.0055). The corresponding mean EQ‐5D utility scores were 0.693 ± 0.031 at stage I, 0.747 ± 0.014 at stage II, 0.762 ± 0.015 at stage III, and 0.750 ± 0.023 at stage IV.

The associations between EQ‐5D utility scores and population characteristics are shown in Table 2. Region (*P* = 0.005), level of education (*P* = 0.031), and age at diagnosis (*P* = 0.041) were significantly associated with EQ‐5D utility scores among patients with esophageal precancerous lesions in univariate analyses. However, the region (*P* < 0.0001), occupation (*P* = 0.021), health care insurance type (*P* = 0.034), pathological type (*P* = 0.0011), type of therapy (*P* < 0.0001), and time points of the survey (*P* < 0.0001) were statistically associated with the EQ‐5D utility scores among cancer patients.

**Table 2:** Mean of EQ‐5D index scores by sociodemographic and clinical characteristics

	Patients with esophageal precancerous lesions		Patients with esophageal cancer	
Variable	Mean (95% CI)	*P*‐value	Mean (95% CI)	*P*‐value
Region				
East	0.885 (0.85–0.92)	0.005*	0.741 (0.73–0.76)	<0.0001*
Central	0.399 (−3.22–4.02)		0.771 (0.75–0.79)	
West	0.813 (0.76–0.86)		0.776 (0.76–0.79)	
Northeast	0.849 (0.79–0.91)		0.662 (0.62–0.70)	
Education				
Primary school or below	0.870 (0.81–0.93)	0.031*	0.756 (0.74–0.77)	0.138
Junior high school	0.818 (0.78–0.86)		0.729 (0.71–0.75)	
Senior high school	0.889 (0.84–0.94)		0.764 (0.74–0.79)	
Undergraduate or over	0.856 (0.77–0.94)		0.738 (0.69–0.79)	
Occupation				
Farmer	0.837 (0.79–0.88)	0.729	0.752 (0.74–0.77)	0.021*
Enterprise or company employee/worker	0.863 (0.82–0.91)		0.779 (0.76–0.80)	
Self‐employed or unemployed	0.851 (0.78–0.93)		0.758 (0.73–0.79)	
Retiree	0.831 (0.72–0.94)		0.758 (0.71–0.81)	
Public sector employee	0.873 (0.81–0.94)		0.697 (0.67–0.73)	
Marital status				
Married	0.853 (0.83–0.88)	0.875	0.751 (0.74–0.76)	0.068
Other	0.850 (0.74–0.96)		0.700 (0.65–0.75)	
Household income in 2012, CNY				
<20 000	0.845 (0.79–0.90)	0.733	0.731 (0.71–0.75)	0.144
20 000–39 999	0.854 (0.76–0.95)		0.751 (0.73–0.77)	
40 000–69 999	0.847 (0.79–0.91)		0.762 (0.74–0.78)	
60 000–79 999	0.848 (0.79–0.90)		0.741 (0.71–0.77)	
≥80 000	0.860 (0.82–0.91)		0.752 (0.72–0.78)	
Health care insurance type				
New rural cooperative medical scheme	0.832 (0.79–0.87)	0.051	0.755 (0.74–0.77)	0.034*
Urban employee basic medical insurance	0.891 (0.85–0.93)		0.746 (0.73–0.77)	
Urban residents basic medical insurance	0.800 (0.72–0.88)		0.711 (0.68–0.74)	
Self‐pay	0.957 (0.41–1.50)		0.831 (0.72–0.94)	
Age at diagnosis, years				
<45	0.890 (0.85–0.93)	0.041*	0.738 (0.67–0.81)	0.909
45–54	0.895 (0.85–0.94)		0.744 (0.72–0.77)	
55–64	0.813 (0.77–0.86)		0.752 (0.74–0.77)	
≥65	0.891 (0.85–0.94)		0.743 (0.73–0.76)	
Pathological type				
Low grade squamous intraepithelial tumors	0.854 (0.81–0.90)	0.7977	—	
High grade squamous intraepithelial neoplasms	0.850 (0.79–0.91)		—	
Adenoma	—		0.723 (0.69–0.76)	0.0011*
Squamous cell carcinoma	—		0.737 (0.72–0.75)	
Other types of cancer	—		0.782 (0.76–0.81)	
Type of therapy				
Surgery	0.885 (0.85–0.92)	0.052	0.661 (0.64–0.68)	<0.0001*
Symptomatic treatment	0.865 (0.83–0.90)		0.734 (0.70–0.77)	
Radiotherapy	—		0.791 (0.77–0.81)	
Chemotherapy	—		0.821 (0.80–0.84)	
Surgery and postoperative chemotherapy	—		0.746 (0.71–0.78)	
Neoadjuvant chemotherapy and surgery	—		0.792 (0.74–0.84)	
Concurrent chemoradiotherapy	—		0.798 (0.77–0.83)	
Other	—		0.804 (0.73–0.88)	
Time points of the survey				
Pretreatment	0.833 (0.78–0.89)	0.217	0.813 (0.79–0.84)	<0.0001*
In treatment	0.838 (0.79–0.89)		0.738 (0.73–0.75)	
Post‐treatment	0.889 (0.85–0.93)		0.736 (0.71–0.76)	
Follow‐up	0.894 (0.84–0.95)		0.720 (0.67–0.77)	

* Significant difference *P* < 0.05.

### Multiple linear regression analyses for EQ‐5D utility scores

Table 3 illustrates the results of multivariable linear regression analyses for EQ‐5D utility scores. Compared with the western region, people from the east or the northeast had higher EQ‐5D utility scores for patients with precancerous lesions, but lower scores for cancer patients. Compared with the new rural cooperative medical scheme, patients who had urban employee basic medical insurance obtained higher EQ‐5D utility scores for patients with precancerous lesions. However, patients who had urban residents' basic medical insurance had lower scores for cancer patients. For patients with esophageal precancerous lesions, the variables of education and age at diagnosis were also retained in the final model (*F* = 3.988, *P* < 0.001, *R* ^2^ = 0.279). Additionally, for cancer patients, the factors of occupation, household income in 2012, pathological type, type of therapy, and time points of the survey were statistically associated with EQ‐5D utility scores in the multivariate analysis (*F* = 11.194, *P* < 0.001, *R* ^2^ = 0.141).

**Table 3:** Multiple linear regression analysis for EQ‐5D index scores

	Patients with esophageal precancerous lesions				Patients with esophageal cancer			
Variable	Coefficient (95% CI)	S.E.	t	*P*‐value	Coefficient (95% CI)	S.E.	t	*P*‐value
Intercept	0.854 (0.77–0.94)	0.044	19.41	<0.001*	0.741 (0.70–0.78)	0.018	39.76	<0.001*
Region (Ref = West)								
East	0.073 (0.02–0.12)	0.026	2.82	0.006*	−0.059 (−0.08–0.03)	0.013	−4.31	<0.001*
Central	0.040 (−0.22–0.30)	0.132	0.30	0.764	−0.005 (−0.03–0.02)	0.015	0.02	0.982
Northeast	0.090 (0.03–0.15)	0.031	2.90	0.004*	−0.126 (−0.16–0.09)	0.018	−6.74	<0.001*
Education (Ref = Primary school or below)								
Junior high school	−0.088 (−0.14− −0.03)	0.028	−3.11	0.002*	—	—	—	—
Senior high school	−0.052 (−0.12– 0.01)	0.034	−1.55	0.124	—	—	—	—
Undergraduate or over	−0.087 (−0.17 − −0.01)	0.040	−2.19	0.030*	—	—	—	—
Occupation (Ref = Farmer)								
Enterprise or company employee/worker	—	—	—	—	0.018 (−0.02–0.06)	0.019	0.93	0.351
Self‐employed or unemployed	—	—	—	—	0.021 (−0.01–0.06)	0.018	1.10	0.269
Public sector employee	—	—	—	—	−0.065 (−0.10– –0.03)	0.019	−3.32	0.001*
Retiree	—	—	—	—	0.013 (−0.04–0.06)	0.025	0.44	0.660
Other	—	—	—	—	0.090 (−0.10–0.28)	0.096	0.95	0.341
Household income in 2012, CNY (Ref = <20 000)								
20 000~	—	—	—	—	0.041 (0.01–0.07)	0.014	3.02	0.003*
40 000~	—	—	—	—	0.049 (0.02–0.08)	0.015	3.32	0.001*
60 000~	—	—	—	—	0.016 (−0.02–0.05)	0.018	1.13	0.259
≥80 000	—	—	—	—	0.033 (0.00–0.07)	0.018	1.96	0.050
Health care insurance type (Ref = New rural cooperative medical scheme)								
Urban employee basic medical insurance	0.077 (0.03–0.13)	0.024	3.16	0.002*	0.008 (−0.03–0.04)	0.018	0.36	0.719
Urban residents basic medical insurance	−0.054 (−0.12–0.01)	0.034	−1.61	0.109	−0.050 (−0.08– ‐0.01)	0.018	−2.60	0.009*
Other insurance	0.117 (−0.07–0.30)	0.093	1.25	0.213	0.005 (−0.05–0.06)	0.030	0.40	0.688
Self‐pay	0.140 (−0.06–0.34)	0.099	1.42	0.159	0.059 (−0.05–0.17)	0.056	1.06	0.288
Age at diagnosis, years (ref = <45)								
45–49	0.021 (−0.05 to 0.09)	0.035	0.58	0.560	—	—	—	—
50–54	−0.066 (−0.13 to 0.00)	0.033	−1.98	0.050	—	—	—	—
55–59	−0.005 (−0.08 to 0.07)	0.037	−0.14	0.887	—	—	—	—
Pathological type (Ref = squamous cell carcinoma)								
Adenoma	—	—	—	—	−0.047 (−0.08– −0.01)	0.019	−2.47	0.014*
Other types of cancer	—	—	—	—	0.040 (0.02–0.06)	0.012	3.41	0.001*
Type of therapy (Ref = symptomatic treatment)								
Surgery	—	—	—	—	−0.065 (−0.10– ‐0.03)	0.017	−3.89	<0.001*
Radiotherapy	—	—	—	—	0.051 (0.01–0.09)	0.019	2.73	0.006*
Chemotherapy	—	—	—	—	0.077 (0.04–0.11)	0.017	4.28	0.000*
Surgery & postoperative chemotherapy	—	—	—	—	0.037 (0.00–0.08)	0.021	1.75	0.080
Neoadjuvant chemotherapy & surgery	—	—	—	—	0.030 (−0.04–0.10)	0.036	0.77	0.442
Concurrent chemoradiotherapy	—	—	—	—	0.066 (0.03–0.11)	0.021	3.23	<0.001*
Other	—	—	—	—	0.039 (−0.06–0.14)	0.051	0.70	0.485
Time points of the survey (Ref = in treatment)								
Pretreatment	—	—	—	—	0.064 (0.04–0.09)	0.015	4.30	<0.001*
Post‐treatment	—	—	—	—	0.027 (0.00–0.05)	0.013	1.84	0.066
Follow‐up	—	—	—	—	0.014 (−0.03–0.06)	0.022	0.56	0.574

Note. Model of patients with esophageal precancerous lesions: F = 3.988 *P* < 0.001 R^2^ = 0.279.

Model of patients with esophageal cancer: F = 11.194 *P* < 0.001 R^2^ = 0.141.

Estimate of partial regression coefficient.

* Significant difference *P* < 0.05.

## Discussion

This current work is a unique large‐scale multicenter study focusing on the HRQoL of patients with EC or precancerous lesions assessed by an internationally comparable and utility instrument, the EQ‐5D questionnaire. Our results provided reliable utility estimates for HRQoL in patients with EC or precancerous lesions using a well‐validated method, which will be informative for future detailed cost‐effectiveness evaluations.

We found that pain/discomfort posed a major problem, followed by anxiety/depression, problem of usual activity, problem of mobility, and problem of self‐care in sequential order. This finding is consistent with the results of another HRQoL analysis of EC using the EQ‐5D instrument in Anhui Province, China.^26^ Chen *et al*. reported the proportion of five dimensions in 209 EC patients in Anhui, where the percentages of respondents reporting problems of pain/discomfort, anxiety/depression, usual activity, mobility, and self‐care were 38.3%, 25.4%, 22.0%, 18.2% and 12.0%.^26^ As expected, there were more cancer patients reporting problems than patients with precancerous lesions. A large‐scale survey, which included the EQ‐5D instrument, was previously conducted based on a national representative sample in 2013 (*N* = 188 720), that was the Chinese National Health Services Survey (NHSS), where the percentages of respondents reporting problems of pain/discomfort, anxiety/depression, usual activity, mobility, and self‐care in the population aged 45–64 years were found to be approximately 14.0%, 5.8%, 3.6%, 4.8%, and 2.3%, respectively.^47^ The current analysis found that 60.5% of cancer patients and 51.3% of patients with precancerous lesions in this survey were suffering pain and discomfort. The proportion was dramatically high when compared with the value of 14.0% of the sampled general population across mainland China.^47^ We also found that a higher proportion of study participants (46.8% of cancer patients and 29.5% of the patients with precancerous lesions) were suffering from anxiety and depression than those in the Chinese general population.^47^ In contrast to the two dimensions mentioned above, the dimensions of self‐care and usual activity in our study population were even more affected by the disease according to comparisons with the results of the Chinese general population.^47^ This situation was due partly to the psychological changes resulting from the function of social role and loss of self‐care ability. During the screening procedure, subjects may experience side effects such as doubt and distress, which will affect their health status. Once the subjects are detected as having cancer, they will be labeled as cancer patients and treated earlier than without screening. Wang *et al*. reported that the worst status of quality of life in cancer patients occurred when patients were receiving treatment.^48^ Therefore, the psychological intervention, including social support and psychological support, is needed to help patients overcome their mental problems.^27^, ^49^

In this study, we used the VAS method to derive values for the health state. In the mentioned NHSS study, the average VAS in the Chinese general population aged 45–64 years was found to be 79.3.^47^ In our study, the measurement of VAS scores suggested that the HRQoL of patients was substantially lowered. As we expected, the VAS score in cancer stage IV was the lowest, which is consistent with the results of the HRQoL analysis in Anhui Province mentioned previously.^26^ Therefore, early diagnosis and treatment of EC is crucial to improving a cancer patient's quality of life. Although the results demonstrated that significant differences of VAS were detected among these age groups of cancer patients, our age curve of VAS scores in patients with EC showed a flat “inverted U" pattern, while the VAS scores of the <45‐year‐old group and ≥ 65‐year‐old group were the lowest. A potential reason for this curve was that young patients with EC are liable to loss of appetite,^50^ whereas young individuals require more energy for fast metabolism, so they rated their health states as low scores. However, with increasing age, the physiological functions^51^ and cognitive functions^52^ of the elderly declined at different levels.

As expected, the cancer patients (0.748) suffered a larger decrease in quality of life than patients with precancerous lesions (0.852). However, there were no significant differences of both EQ‐5D utility scores and VAS scores between males and females, which suggested that sex was not an influencing factor of HRQoL. The scores differed notably among cancer stages. There is some debate about the association between EQ‐5D utility score and the stage of EC. It was surprising to see that the utility score for cancer stage I was the lowest, and it was inconsistent with the results of previous studies.^26^, ^27^, ^53^ For example, Wildi *et al* reported that the utility scores were negatively associated with the severity of EC.^54^ However, as for other types of cancer, Wong *et al*. reported worse HRQoL in patients with earlier stage of colorectal cancer compared with those at stage III and IV.^55^ Thus, further studies are needed to explore the relationship between utility score and cancer stage.

For patients with EC, compared with the western region, people from the east or the northeast had lower EQ‐5D utility scores, inconsistent with the results mentioned above.^47^ In China, the socioeconomic status of eastern areas is better than western areas. Previous studies have suggested that respondents with lower socioeconomic status might have lower expectations of health, and under the same health conditions, they might assess their own health status as higher than respondents in a higher socioeconomic status.^56^ It is not surprising that patients with higher household incomes tended to have higher scores. Patients who owned urban resident basic medical insurance had lower scores in comparison with the patients who owned new rural cooperative medical insurance. Such findings were consistent with the results of the previous HRQoL studies in China.^26^, ^57^ The New Rural Cooperative Medical Scheme is a main medical security form of Chinese rural citizens, which has made remarkable achievements since 2003 when it was initiated, and many insurers have received benefits from it.^58^ Zhou *et al*. have also reported that having new rural cooperative medical insurance significantly improved the HRQoL of residents.^59^ We concluded that patients with adenoma received lower scores, compared with squamous cell carcinoma. Studies have indicated that the prognosis of adenoma is poorer than that of squamous cell carcinoma.^60^ Our findings also suggested the type of therapy might impact the HRQoL of patients. Patients who underwent surgery received the lowest EQ‐5D utility scores, while patients who received chemotherapy, radiotherapy, and concurrent chemoradiotherapy received higher scores than those who received symptomatic treatment. It was identical to the results of the HRQoL analysis in Anhui Province mentioned previously.^26^ Lagergren *et al*. reported that esophagectomy for cancer had a temporary negative impact on most aspects of self‐reported HRQoL, which typically recovered within the first postoperative year.^61^ We conducted this cross‐sectional survey just one or two years after cancer screening, so surgery had a negative influence on the HRQoL. The time points of this survey were also an important influencing factor. The participants were interviewed at one of the following four time points: pretreatment (before the commencement of the treatment process); in‐treatment (under the treatment process); post‐treatment (participants who had completed the main treatment process and were soon leaving hospital); follow‐up (more than one month after the end of treatment). It is understandable that patients who completed the survey before treatment had higher scores than those in treatment. In a previous study, persistent deterioration in physical function and increased breathlessness, diarrhea, and reflux were observed during treatment and within 6–12 months after surgery.^61^

For patients with esophageal precancerous lesions, our finding that the western patients suffered lower EQ‐5D utility scores than the eastern and northeastern patients could be explained by the unequal economic development between China's eastern and western areas. The economic conditions and healthcare services in the western areas lag behind the eastern areas because of the limitations of geographical location. Our results suggested that patients with diplomas of junior high school or undergraduate or above had lower EQ‐5D utility scores that patients with diplomas of primary school or below, which may be because the highly‐educated patients suffered higher stress from jobs and from living a faster‐paced life.

The ability to place a value on the physical and emotional experiences of patients undergoing screening is important in the quality‐adjustment of any survival advantage that might be afforded by cancer screening.^62^ Our findings determined the underlying relevant factors that impacted the HRQoL or utility values of patients, which will be informative for future EC prevention and control efforts.

One potential limitation is that this study was a cross‐sectional survey rather than a longitudinal study. It did not provide a comprehensive description of the temporal trend of HRQoL. However, as a key part of the CanSPUC, we will continue with the long‐term follow‐up survey in subsequent years. In addition, EQ‐5D‐5L, a new version of the EQ‐5D questionnaire has been developed with a higher sensitivity,^63^ and the Chinese value set of EQ‐5D‐5L was published in 2017.^64^ The comparison between the three‐level and five‐level instruments is worth noting.^65^

In conclusion, the section of our study based on the EQ‐5D instrument substantially provided the detailed HRQoL utility values of EC patients across mainland China, which will be informative for future cost‐utility analyses. We also provided information about the variables affecting the HRQoL of EC patients. Psychological intervention, social support, early diagnosis, and early treatment, and critical illness insurance are needed to help to improve the quality of life in patients. The findings of influencing factors are beneficial for future EC prevention and control efforts.

## Disclosure

The authors confirm that there is no conflict of interest.
